# Immunosuppressive Effect of *Litsea cubeba* L. Essential Oil on Dendritic Cell and Contact Hypersensitivity Responses

**DOI:** 10.3390/ijms17081319

**Published:** 2016-08-12

**Authors:** Hsin-Chun Chen, Wen-Te Chang, You-Cheng Hseu, Hsing-Yu Chen, Cheng Hsuan Chuang, Chi-Chen Lin, Meng-Shiou Lee, Ming-Kuem Lin

**Affiliations:** 1 Department of Cosmeceutics, College of Biopharmaceutical and Food Sciences, China Medical University, No. 91, Hsueh-Shih Road, Taichung 40402, Taiwan; c0706@mail.cmu.edu.tw (H.-C.C.); ychseu@mail.cmu.edu.tw (Y.-C.H.); 2 Department of Chinese Pharmaceutical Sciences and Chinese Medicine Resources, College of Biopharmaceutical and Food Sciences, China Medical University, No. 91, Hsueh-Shih Road, Taichung 40402, Taiwan; wtchang@mail.cmu.edu.tw (W.-T.C.); cappuccino2027@hotmail.com (H.-Y.C.); zhushaun920@gmail.com (C.H.C.); 3 Institute of Medical Technology, College of Life Science, National Chung Hsing University, Taichung 402, Taiwan; lincc@dragon.nchu.edu.tw

**Keywords:** *Litsea cubeba*, essential oil, dendritic cell, immunosuppressive, citral

## Abstract

*Litsea cubeba* L., also named as Makauy, is a traditional herb and has been used as cooking condiment or tea brewing to treat diseases for aborigines. The present study was undertaken to explore the chemical compositions of the fruit essential oil of *L. cubeba* (LCEO) and the immunomodulatory effect of LCEO on dendritic cells and mice. The LCEO was analyzed using gas chromatography (GC) and gas chromatography/mass spectrometry (GC/MS) with direct injection (DI/GC) or headspace-solid phase microextraction (HS-SPME/GC). In total, 56 components were identified, of which 48 were detected by DI/GC and 49 were detected by HS-SPME/GC. The principal compounds were citral (neral and geranial). An immunosuppressive activity of LCEO was investigated with bone marrow-derived dendritic cells (DCs) which have a critical role to trigger the adaptive immunity. Additionally, the inhibitory effect of LCEO on immune response was elucidated by performing the contact hypersensitivity (CHS) responses in mice. Our results clearly showed that LCEO decreases the production of TNF-α and cytokine IL-12 in a dose-dependent manner in lipopolysaccharide (LPS)-stimulated DCs. CHS response and the infiltrative T cells were inhibited in the tested ears of the mice co-treated with LCEO. We demonstrate, for the first time, that the LCEO mainly containing citral exhibits an immunosuppressive effect on DCs and mice, indicating that LCEO can potentially be applied in the treatment of CHS, inflammatory diseases, and autoimmune diseases.

## 1. Introduction

In the immune system, various immune cells are highly communicative with each other by various cytokines and are in charge of the defense against foreign pathogen infection and maintaining health. Inflammation is a major component of our immune response. Although inflammation is a natural defense, the persistence of the process for abnormally long periods can be harmful and has been recognized as a major risk factor for various human diseases, including cardiovascular disease, metabolic disorder, neurological disease, and cancer [1,2]. Thus, reduction of chronic inflammation would be beneficial to prevent the pathological progression of these human diseases. In such cases, the inflammatory response should be suppressed. Among the immune cells, dendritic cells (DCs) are the best antigen-presenting cells and are in charge of the induction of adaptive immunity [3,4,5]. To initiate adaptive immunity, DCs present a specified antigen on the surface to be recognized by naïve T cells. This recognition triggers the differentiation of the specified antigen-specific T cells. Next, a strong and specific T cell-based immune response is built up to attack the “pathogens” which present the specified antigen. With such a critical role, DCs are thought to be an ideal target when attempting to evaluate potential immune modulators [6,7,8].

*Litsea cubeba* L. belongs to the family Lauraceae, which is widely distributed in Japan, Taiwan, Southern China, and Southeastern Asia [9]. All parts of this plant emanate a pungent gingery odor [10]. Fruits of *L. cubeba* are spicy condiments, frequently used in the aboriginal cuisine of Taiwan [11]. The essential oil of its fruit has been used as a flavor enhancer in cigarettes, cosmetics, and foods, and as raw material to produce citral (neral and geranial) [12]. Its pharmacological effects have been reported to have an antimicrobial [9,13], antioxidative [14], anticancer [10,15], anti-inflammatory [11], and insecticidal activities [12,16]. However, there is no report on immunosuppressive effects of *L. cubeba* on DCs and its relative immune response in vivo. In this study, *L. cubeba* essential oil (LCEO) was extracted from the fresh fruits. The inhibitory effect of the LCEO on dendritic cell activation was examined. In addition, the contact hypersensitive response was conducted to examine the in vivo immunosuppressive effects in mice. 

## 2. Results and Discussion

### 2.1. Constituents of the Essential Oils

Punyarajun and Nandhasri (1981) extracted essential oils from unripe *L. cubeba* berries of Thai origin and the yield was ca. 3.0% [17]. Ho et al. (2010) used hydrodistillation to extract the leaf and fruit essential oils of *L. cubeba* from Taiwan, and the yields were 13.9% ± 0.09% and 4.0% ± 0.03%, *v*/*w*, respectively [10]. Liu and Yang (2012) extracted the fruit essential oils of *L. cubeba* from Taiwan, and the yields were 4.5% ± 0.2% [9]. Jiang et al. (2009) reported that the fruit of *L. cubeba* contains 3%–5% of essential oils which are rich in citral [12]. In the present study, the yield of the essential oils obtained from the fresh fruit of *L. cubeba* by steam distillation was 3.7% ± 0.4%. The yield is similar to that reported in these published studies.

As shown in Table 1, a total of 48 components were identified by gas chromatography using direct injection (DI/GC). These components include 12 monoterpenes, five sesquiterpenes, seven terpene alcohols, three terpene aldehydes, two terpene ketones, six terpene esters, five terpene oxides, four aliphatic aldehydes, one aliphatic ketone, and three aliphatic ester. The principal compounds were citral (neral and geranial) accounting for 88.02%. Other constituents identified in significant proportions were 6-methyl-5-hepten-2-one, β-myrcene, limonene, linalool, citronellal, and verbenol. Terpene aldehydes (89.25%) were the most abundant compounds in the essential oil (Table 1). In line with the studies from Seo et al. [16], Ho et al. [10], Kejlová et al. [18], Liu and Yang [9], this study showed that terpene aldehydes were the most abundant volatile compounds and neral and geranial were the most major components in the oil.

The headspace-solid phase microextraction (HS-SPME) method has been reported to be an excellent tool for the analysis of herbs because it is simple, fast, and does not leave any residues [19]. In this study, a total of 49 components were identified by GC and GC/MS with HS-SPME method. These components include 15 monoterpenes, five sesquiterpenes, seven terpene alcohols, three terpene aldehydes, three terpene ketones, seven terpene esters, six terpene oxides, four aliphatic aldehydes, one aliphatic ketone, one aliphatic alcohol, and three aliphatic esters. Terpene aldehydes (75.09%) were the most abundant compounds in the oils (Table 1).

Comparative analysis of these compounds identified by these two methods, showed that 56 compounds were detected in total, of which, 48 were identified by DI/GC and 49 by HS-SPME/GC. As shown in Table 1, HS-SPME/GC analysis revealed higher percentages of monoterpenes, terpene alcohols, and aliphatic ketone, but lower percentages of terpene aldehydes and terpene esters than DI/GC analysis. Some monoterpenes such as α-thujene, α-phellandrene, and trans-β-ocimene could be identified only by HS-SPME/GC. This indicates that the more complete constituents of essential oils can be identified with the combination of DI/GC and HS-SPME/GC. 

### 2.2. L. cubeba Essential Oils (LCEO) Inhibited the Activation of Dendritic Cells (DCs)

**Figure 1:**
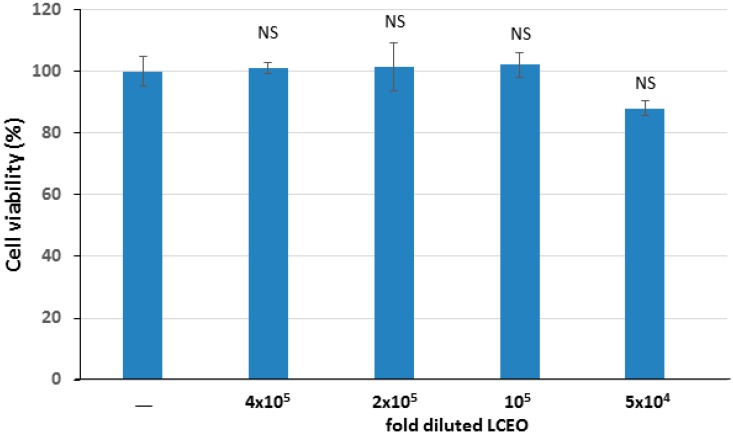
*L. cubeba* essential oils (LCEO) did not impair cell viability of dendritic cells (DCs). DCs were treated with LCEO at different concentrations (5 × 10^4^-, 1 × 10^5^-, 2 × 10^5^- and 4 × 10^5^-fold dilutions) at 37 °C in 5% CO_2_/air for 24 h. The cytotoxicity of LCEO was examined by Cell Counting Kit-8 (CCK-8) assay (Sigma-Aldrich, St. Louis, MO, USA). ^NS^ *p* > 0.05 (Mann–Whitney *U*-test) for the comparison between LCEO-treated and untreated DCs.

**Figure 2:**
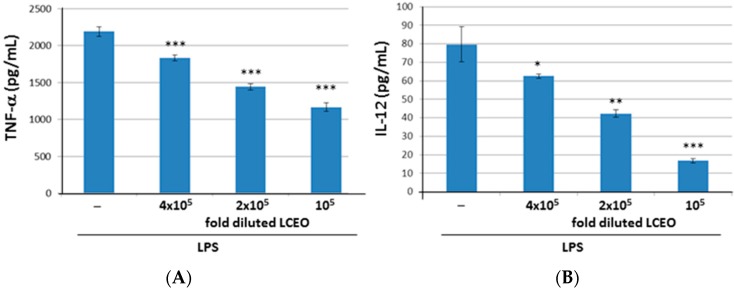
The release of TNF-α (**A**) and IL-12 (**B**) by lipopolysaccharide (LPS)-induced DCs were inhibited by LCEO. The DCs were treated with LPS or LPS + LCEO at different concentrations (1 × 10^5^-, 2 × 10^5^- and 4 × 10^5^-fold dilutions). Supernatants were collected after 6 h to detect TNF-α and 24 h to detect IL-12. The amounts of TNF-α and IL-12 were determined by enzyme-linked immuno sorbent assay (ELISA). Each value represents the mean ± SD (standard deviation) of the data obtained from three wells for each treatment. * *p* < 0.05, ** *p* < 0.01 and *** *p* < 0.001 (Mann–Whitney *U*-test) for the comparison between the LCEO treated LPS-activated DC groups and the untreated LPS-activated DC group. All data are representative of three independent experiments.

To test that the cytotoxic effect of *L. cubeba* essential oils (LCEO) on dendritic cells (DCs), the viability of mouse bone marrow-derived DCs treated with the different concentrations of LCEO was examined. The result showed no significant effect of 5 × 10^4^-, 1 × 10^5^-, 2 × 10^5^- and 4 × 10^5^-fold diluted LCEO on DCs, although 5 × 10^4^-fold diluted LCEO exhibited a little cytotoxic effect (Figure 1). Thus, the concentrations of 1 × 10^5^-, 2 × 10^5^-, and 4 × 10^5^-fold diluted LCEO was used for the following inhibition experiment. TNF-α and IL-12 are hallmarks of DC activation [3,6,7,8]. To elucidate the immunomodulatory activity of the LCEO, the effect of LCEO on TNF-α and IL-12 production by DCs stimulated by lipopolysaccharides (LPS) was examined. The results showed that the amounts of TNF-α and IL-12 produced by LPS-induced DCs were inhibited by the presence of LCEO in a dose-dependent manner (Figure 2). This indicated that LCEO possess an inhibitory activity to DC activation. The IC_50_ of LCEO for TNF-α and IL-12 was approximately 1 × 10^5^- and 2 × 10^5^-fold dilution, respectively.

### 2.3. The Contact Hypersensitivity (CHS) Response Is Attenuated in Mice Co-Treated with LCEO

**Figure 3:**
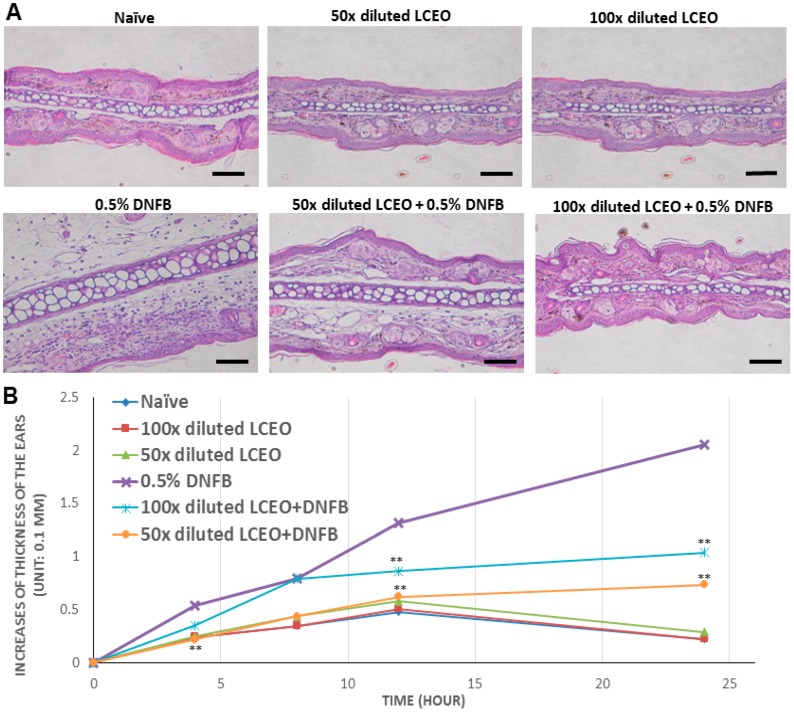
The contact hypersensitivity (CHS) response was attenuated in mice that had been treated with LCEO. 2,4-Dinitro-1-fluorobenzene (DNFB)-induced hypersensitivity response was carried out as described in the “Materials and Methods”. Mice were sensitized with vehicle (blue), 0.5% DNFB (purple), 100-fold diluted LCEO (red), 50-fold diluted LCEO (green), 0.5% DNFB + 100-fold diluted LCEO (light blue), or 0.5% DNFB + 50-fold diluted LCEO (orange) by painting their abdomens. The hypersensitivity response was examined by histological analysis using hematoxylin and eosin staining (**A**), and by measuring the thickness of the tested ear at 4, 8, 12, and 24 h (**B**). The scale bar represents 0.2 mm. Each value represents as mean ± SD from data of each group. ** *p* < 0.01 (Mann–Whitney *U*-test) for the comparison between the LCEO-treated DNFB-sensitized mouse group and the untreated DNFB-sensitized mouse group.

**Figure 4:**
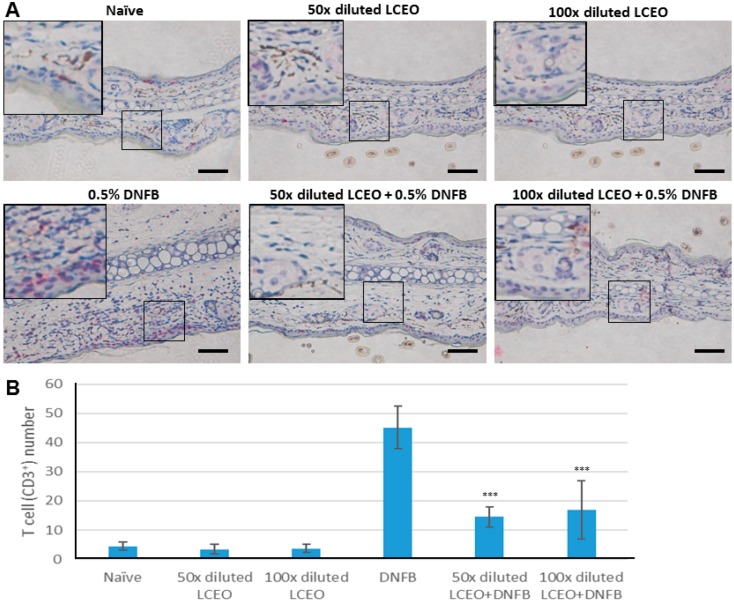
Infiltrative CD3^+^ cells were significantly reduced in the tissue of the tested ears treated with LCEO. (**A**) CD3^+^ cells were detected by CD3 immunohistochemistry in the tissue of the tested ears. The scale bar represents 0.2 mm; (**B**) The number of CD3^+^ cells in ten immunostained tissue samples from the tested ears of each group was determined by manually counting the number of red cells (CD3^+^) under a light microscope. The counts were summarized and then used to make the plot. Each value represent as the mean ± SD. *** *p* < 0.001 (Student’s *t*-test) for comparison with the untreated DNFB-sensitized mouse group.

The above findings indicate that LCEO is able to inhibit the activation of DCs and, thus, we are able to postulate logically that LCEO is able to prevent DC-mediated immune response. Therefore, DNFB-induced CHS was performed to examine the inhibition as the immune response stimulated by DNFB is a type of cell-mediated response. Mice were sensitized by painting their abdomens with DNFB in the absence or presence of LCEO. The hypersensitivity response to DNFB at the ears was then examined. The results of the histological analyses (Figure 3A) and the increase of thickness of the tested ears (Figure 3B), showed that the tested ears were significantly swollen in DNFB-sensitized mice but not in DNFB plus LCEO-treated mice (by painting), indicating that LCEO significantly inhibits the CHS in the DNFB-sensitized mice. Moreover, CD3^+^ T cells, which are activated by DC cells, were examined by immunostaining analysis in the tissue of the tested ears. The results showed that infiltrative T cells are significantly reduced (Figure 4A). To quantify the inhibitory effect, the number of the infiltrative T (CD3^+^) cells in the tested ears was counted. The results showed that infiltrative CD3^+^ cells were significantly reduced by the presence of LCEO (Figure 4B). Collectively, these results provided evidence that LCEO have the potential to prevent or treat delayed-type hypersensitivity/type-4 hypersensitivity; for example, allergic contact dermatitis.

By chemical analysis, we found that neral and geranial were the most common components. Liao et al. separated citral into neral and geranial in pure forms and demonstrated their anti-inflammatory activity, and neral showed a greater anti-inflammatory activity, including significant inhibition of cytokine secretion and inflammatory molecule expression of LPS-stimulated macrophages [11]. Therefore, it is likely that neral and geranial can be the major active constituents in LCEO which contribute to the immunosuppressive effects exhibited in the present study.

Increasingly, recent research has focused on identifying immune modulators in native resources, particularly in edible material. The reason is that the compounds in such materials are relatively safe to humans and, thus, may be regarded as safe immune modulators. *L. cubeba* has long been used to treat various diseases and as a functional food for aborigines, thus can be taken as a good native resource candidate. In this study, LCEO extracted from *L. cubeba* fruits was shown to possess immunomodulatory activity, as seen by the immunosuppressive activity to DCs in DNFB-sensitized mice. Therefore, the in vitro and in vivo results revealed that LCEO has the ability to inhibit hypersensitivity responses by affecting DC functioning. Moreover, DCs play a role to develop chronic inflammation and autoimmunity [20,21]. Thus, we have provided, for the first time, evidence that LCEO may be a promising agent for the treatment of inflammation and autoimmune diseases.

## 3. Materials and Methods

### 3.1. Plant Material

Fresh *Litsea cubeba* fruits were collected from a spicebush farm Wanrong Township, Hualien, Taiwan. These fruits were washed using running water and then air-dried at room temperature overnight.

### 3.2. Methods

#### 3.2.1. Preparation of *L. cubeba* Essential Oil

Fresh fruits of *L. cubeba* (400 g) were homogenized for 2 min with 1200 mL of deionized water. The homogenate was put into a 5 L round-bottom flask and steam-distilled for 4 h to extract the essential oils. The oil was dried over anhydrous sodium sulfate. The prepared samples were immediately stored in brown flasks at −20 °C (freezer) prior to analyses by gas chromatography (GC) and bioassays.

#### 3.2.2. Analysis of the Volatile Constituents

(1) Direct injection analytic method (DI): 1 μL of essential oil was injected into the gas chromatograph injection unit. All experiments in the present study were performed in triplicate.

(2) Headspace-solid phase microextraction (HS-SPME) analysis: A 50/30 μm divinylbenzene/carboxen/polydimethylsiloxane fiber (Supelco, Inc., Bellefonte, PA, USA) was exposed to each sample (1 mL) as placed in a 22 mL vial (precleaned # 27343 clear screw cap vials; Supelco, Bellefonte, PA, USA) for 20 min at 25 °C; the fiber was then injected into the gas chromatograph injection unit.

(3) Analysis of GC: quantitative analyses of the volatile compounds were performed using an Agilent 7890A GC (Santa Clara, CA, USA) equipped with a DB-1 (60 m × 0.25 mm i.d., 0.25 μm film thickness) fused-silica capillary column with a flame ionization detector. The oven temperature was held at 40 °C for 1 min and then raised to 150 °C at 2 °C/min and held for 1 min, and then increased from 150 to 200 °C at 10 °C/min and held for 3 min. Injector and detector temperatures were maintained at 250 °C and 300 °C, respectively. The nitrogen gas flow rate was 1 mL/min. Kovats indices were calculated for the separated components relative to a C5-C25 n-alkanes mixture [22]. The method used was modified as previously described [23].

(4) Analysis of GC-MS: qualitative analyses of volatile compounds were identified using an Agilent 7890B GC (Santa Clara, CA, USA) equipped with a DB-1 (60 m × 0.25 mm i.d., 0.25 μm film thickness) fused-silica capillary column coupled to an Agilent model 5977 N MSD mass spectrometer (MS) (Agilent model 5977 N MSD mass spectrometer). The GC conditions in the GC-MS analysis were the same as in the GC analysis. The injector temperature was maintained at 250 °C. The helium gas flow rate was 1 mL/min. The electron energy was 70 eV at 230 °C. The constituents were identified by matching their spectra with those recorded in an MS library (Wiley 7n). The constituents were confirmed by comparing the Kovats indices or GC retention time data with data published in the literature or those of authentic standards. The method used was modified as previously described [23].

### 3.3. Preparation of Mouse Bone Marrow-Derived Dendritic Cells

C57BL/6 mice, which were purchased from Taiwan, were used in this study. All animals were housed in a specific pathogen-free facility in the Division of Laboratory Animals, China Medical University. All mice were maintained and handled according to standard protocols and the protocols was approved (103-156-N, 27 December 2012) by the Institutional Animal Care and Use Committee, China Medical University. The bones of mice were collected and bone marrow-derived dendritic cells (DCs) were prepared as previously described [6,7,8].

### 3.4. Cytotoxicity Assay of LCEO

The cytotoxicity of LCEO was examined by Cell Counting Kit-8 (CCK-8) assay (Sigma-Aldrich, St. Louis, MO, USA). The LCEO was diluted into 50-fold diluted stock with dimethyl sulfoxide. The DCs were treated with LCEO at different concentrations (5 × 10^4^-, 10^5^-, 2 × 10^5^-, and 4 × 10^5^-fold dilution in final) at 37 °C in 5% CO_2_/air for 24 h. The cells were then harvested and the viability measured according to manufacturer’s instruction.

### 3.5. Measurement of Cytokines Production by DCs

Cytokine production was measured by enzyme-linked immuno sorbent assay (ELISA) as described previously [6,7,8]. The DCs were treated with lipopolysaccharide (LPS, 100 ng/mL) from *Escherichia coli* 055:B5 (Sigma) or LPS + LCEO (5 × 10^4^-, 1 × 10^5^-, and 2 × 10^5^-fold dilution in final) for 6 h for TNF-α and 24 h for IL-12. The production of TNF-α and IL-12p70 was measured using the ELISA kit (eBioscience, San Diego, CA, USA). 

### 3.6. The Assay of Contact Hypersensitivity (CHS) Response 

2,4-Dinitro-1-fluorobenzene (DNFB; Sigma-Aldrich, St. Louis, MO, USA)-stimulated hypersensitivity was conducted as previously described [8,24]. Briefly, 12 mice were used and grouped into four groups. To bring about sensitization, their abdomens were painted with vehicle, DNFB, 50-fold diluted LCEO, DNFB + 50-fold diluted LCEO, or DNFB + 100-fold diluted LCEO every day for 5 days. Then, both ears of all mice were painted with DNFB on the sixth day. The phenotype of the CHS were determined histologically in 24 h using hematoxylin and eosin staining. The thickness of the tested ear were measured. The increase of the thickness was calculated by the thickness of the challenged ear minus the thickness of the unchallenged ear. By immunostaining analysis using anti-CD3 antibody, the number of infiltrating T cells in the tested ear was detected and calculated as previously described [8]. 

### 3.7. Data Analysis

In order to assess the significance of the differences in the levels of the cytokines and the increase of thickness of ear, the Mann–Whitney *U*-test was used. In order to assess the significance of the differences in the numbers of CD3^+^ T cells, the Student’s *t*-test with a two-tailed distribution and two-sample equal variance was used. Values of ** *p* < 0.01 and *** *p* < 0.001 were considered highly significant. A value of * *p* < 0.05 was considered significant.

## 4. Conclusions

A total of 56 components were identified in LCEO. Forty-eight were detected by DI/GC, and 49 were detected by HS-SPME/GC. The principal compounds were neral and geranial (citral). LCEO inhibits DC functioning. Thus, LCEO may be useful in the treatment of inflammatory diseases.

## Figures and Tables

**Table 1:** Means of volatile compounds in *Litsea cubeba* essential oils (LCEO) analyzed by gas chromatography with direct injection (DI/GC) and headspace-solid phase microextraction (HS-SPME/GC).

Compound	RI ^z^	Content (%) ^y^	
		DI/GC	HS-SPME/GC
**Monoterpenes**		4.01	11.91
α-thujene	921	– ^w^	0.02
α-pinene	931	0.22	0.72
camphene	945	0.03	0.10
sabinene	972	0.07	0.06
β-pinene	976	0.09	0.20
β-myrcene	980	0.77	2.11
α-phellandrene	998	–	0.01
α-terpinene	1007	<0.01	–
ρ-cymene	1014	0.01	0.01
limonene	1026	2.73	8.50
*cis*-β-ocimene	1026	0.01	0.02
*trans*-β-ocimene	1032	–	0.02
γ-terpinene	1050	0.01	0.01
α-terpinolene	1078	0.04	0.11
1,3,8-ρ-menthatriene	1094	0.03	0.02
**Sesquiterpenes**		0.10	0.06
α-copaene	1366	0.01	0.01
β-elemene	1382	0.04	<0.01
β-caryophyllene	1429	0.03	0.04
α-humulene	1441	0.02	0.01
δ-cadinene	1525	<0.01	<0.01
**Terpene alcohols**		2.75	5.22
linalool	1079	1.23	1.11
isopulegol	1128	0.03	–
verbenol	1130	1.31	3.81
α-terpineol	1183	0.07	0.06
*cis*-carveol	1189	0.07	0.19
*cis*-geraniol	1237	0.03	0.05
nerolidol	1558	0.01	–
**Terpene aldehydes**		89.25	75.09
citronellal	1127	1.23	1.63
neral	1226	38.02	34.17
geranial	1256	50.00	39.29
		(2854.05 mmol/L)	
**Terpene ketone**		0.14	0.10
camphor	1113	0.14	0.04
piperitone	1230	<0.01	<0.01
piperitenone	1308	–	0.06
**Terpene ester**		0.32	0.14
methyl salicylate	1163	0.05	0.01
bornyl acetate	1286	0.01	0.02
terpinenyl acetate	1335	0.07	0.02
citronellyl acetate	1357	0.02	0.02
geranyl acetate	1362	0.16	0.06
neryl acetate	1366	0.01	0.01
methyl cinnamate	1384	–	<0.01
**Terpene oxide**		0.16	0.17
1,8-cineole	1019	0.12	0.14
*trans*-linalool oxide	1055	<0.01	0.02
*cis*-rose oxide	1086	<0.01	<0.01
*trans*-rose oxide	1089	–	<0.01
limonene oxide	1128	0.01	–
caryophyllene oxide	1571	0.03	0.01
**Aliphatic aldehydes**		0.01	0.03
3-methyl butanal	631	<0.01	–
2-methyl butanal	636	<0.01	–
pentanal	697	–	<0.01
hexanal	776	<0.01	0.01
2,6-dimethyl hept-5-enal	1047	0.01	0.02
**Aliphatic ketone**		1.19	2.23
6-methyl-5-hepten-2-one	962	1.19	2.23
**Aliphatic alcohol**		–	<0.01
2-methyl-3-buten-2-ol	600	–	<0.01
**Aliphatic esters**		0.01	0.01
ethyl isovalerate	825	<0.01	–
isoamyl acetate	864	0.01	0.01
ethyl tiglate	915	<0.01	<0.01

^Z^ Retention indices, using paraffin (C_5_-C_25_) as references; ^y^ Values are means of triplicates; ^w^ undetectable.
